# The PB1-F2 protein of Influenza A virus: increasing pathogenicity by disrupting alveolar macrophages

**DOI:** 10.1186/1743-422X-4-9

**Published:** 2007-01-15

**Authors:** J Robert Coleman

**Affiliations:** 1 Department of Molecular Genetics and Microbiology, Stony Brook University, Stony Brook, NY 11794, USA

## Abstract

With the prospect of another pandemic Influenza fresh in our consciousness, the pathogenic nature of the Influenza A virus and its ability to induce high rates of mortality are ever more pertinent. Recently a novel protein encoded by an alternate reading frame in the PB1 Gene segment of Influenza A virus has been discovered and in turn shown to enhance viral virulence in a mouse model [1]. This protein has been shown to specifically target and destroy alveolar macrophages [2]. This review suggests that this protein, present in all previous pandemic strains, may reappear as a virulence factor in a subsequent pandemic strain. This PB1-F2 protein will enhance the mortality rate of the virus by increasing the likelihood of a secondary bacterial infection, which is the primary cause of death to a patient infected with Influenza A.

## Background

An intrinsic property of all viruses is their need to either subvert and/or subdue the host immune response to establish a productive replication cycle. The mechanism by which this avoidance proceeds is greatly varied throughout the phyla of viruses; however either must occur in order for a virus to spread and propagate. The enhancement of this ability to subvert and disable host immunity is directly correlated to increased viral pathogenicity in the host [3]. For example the 3C viral protease of Poliovirus cleaves the p65-RelA subunit of NF-κB, a transcription factor involved in the cellular innate immune response [4]. Another human pathogen, Influenza A virus, has many well-elucidated mechanisms used to avoid both the innate and adaptive human immune responses. Since the current concern over the possible emergence of an Influenza A virus pandemic has arisen, Influenza A virus's mechanisms of evasion are of great significance. The main culprit of evasion on the cellular level is the NS1 protein, which is responsible for inactivation of the host innate immune response by preventing activation of PKR from INF-α/β signaling, thus allowing replication and viral protein synthesis to proceed unabated in the host cell [5]. Additionally, Influenza A virus has the ability to escape the host's humoral immunity by a phenomenon known as antigenic drift. This mutation driven phenomena produces changes both in amino acids and glycosylation patterns of the two virus envelope glycoproteins, hemagglutinin (HA) and neuraminidase (NA) [6]. This concept of antigenic drift is significant because it contributes to the continued, seasonal pathogenicity associated with epidemic Influenza A virus, i.e. the need for a new flu vaccine each year. Since Influenza manipulates and creates new antigenic determinates via mutations, the human population becomes steadily immunologically inept, where by circulating antibodies are incapable of, or have a reduced capacity to, neutralize a repeat infection. A more threatening avoidance of humoral and innate cellular immunity is the ability of Influenza A virus to reassort via the phenomena of antigenic shift. Since Influenza A virus is an eight-segmented minus-sense RNA virus, its segmented nature allows for the swapping and exchange of gene segments between different strains. Specifically this occurs by human influenza viruses swapping the HA glycoprotein, NA glycoprotein or polymerase (PB1, PB2, PA) segments with those of avian and pig Influenza A viruses. Therefore, a reassortment of gene segments has occurred creating an entirely novel Influenza A virus strain capable of infecting humans. [6]. In turn, the global human population is entirely immunologically naïve to these novel viruses and these viruses become the cause of pandemics that result in a vast number of human deaths; the most notably of which was the 1918 Spanish Flu that killed an estimated 30 to 50 million people [7].

### Influenza A virus and the PB1-F2 protein

Other additional mechanisms of host defense manipulation and avoidance by Influenza A virus exist including a novel alternate reading frame recently discovered in the PB1 polymerase gene segment. This reading frame is found in select Influenza A viruses and has been shown to impact host defense mechanisms and in turn enhance pathogenicity in vivo [1]. This protein, named PB1-F2, has an apoptotic induction effect on macrophages, thus reducing their ability to contribute to an immune response [8]. It has been previously suggested that this PB1-F2 protein contributes to viral pathogenicity solely because of it causes an inhibition of viral clearance, thus increasing cytoxicity [1]. However, it is possible that by directly targeting professional antigen presenting cells for destruction, the PB1-F2 can also contribute to Influenza A virus pathogenicity by increasing the probability of an opportunistic secondary bacterial infection. The major source of mortality associated with Influenza A infection is indeed these secondary bacterial infections [9].

The discovery of a novel protein that contributes to an increase in Influenza A virus pathogenicity is of great concern due to the impact that Influenza A virus has on both human health as well as on the economy. Globally it is estimated that between 300,000 to 500,000 people may die annually due to influenza virus infections [10]. In the United States, influenza viruses are the cause of widespread mortality and morbidity. For example, influenza viruses cause approximately 35,000 deaths each year in the United States [11]. In addition to the impact on human health, influenza virus infections are responsible for an estimated 200,000 hospitalizations, thus impacting the United States economy by an approximate cost of 23 billion dollars each year [12]. Although a productive viral infection can elicit disease in all age groups, serious, life-threatening complications are markedly increased in children and elderly persons 65 years of age and over. By further elucidation of the various mechanisms that contribute to the pathogenic nature of Influenza A virus, there can be a beneficial impact on human health and the economy by way of enhancing vaccine development or increasing the effectiveness of antiviral drugs.

Specifically, Influenza A virus is an enveloped, eight segmented minus-sense RNA virus, that is a member of the orthomyxoviridae genus of viruses. Unique to Influenza A virus, compared to other lytic mammalian RNA viruses, is that all mRNA synthesis and genome replication occurs in the nucleus of the infected host cell [13]. Furthermore, Influenza A virus replication proceeds rapidly, evident by the completion of a replication cycle in 10 hours. Primarily Influenza A virus infects the respiratory tract via the inhalation of aerosols containing infectious virions. The infection and replication of Influenza A virus is cytolytic and is usually limited to superficial epithelial cells of the upper and lower respiratory tract. However, secondary sites of infection can occur, such as infections of nervous tissue, which results in Reye's syndrome- a condition characterized by acute encephalopathy [14].

### PB1-F2 aiding in secondary bacterial infections?

Despite Influenza A virus's speedy replication cycle and cytolytic properties, the major cause of death associated with an Influenza A virus infection is secondary bacterial pneumonia. The major pathogens associated with this complication are *Streptococcus pneumoniae*, *Staphylococcus aureus*, and *Haemophilus influenzae *[9]. One of the symptoms usually associated with flu, dry cough, is indicative of the loss of ciliated, mucous-producing epithelial cells as a result of the viral infection destroying these cells lining the respiratory tract [15]. The mucus produced by these cells in a normal, healthy individual serves to clear invading microorganisms as well as particulate matter [16]. However, during an Influenza A virus infection this clearance function is significantly impaired and therefore opportunistic bacteria can reside in the lung longer, and in turn establish a secondary infection which results in the increased death rate.

Besides the destruction of mucus producing lung epithelia cells, another possible contribution to secondary bacterial infection as the result of Influenza A infection is the targeted elimination of alveolar macrophages by the Influenza PB1-F2 protein. Initially precursors to macrophages, monoblasts, are produced in the bone marrow and secreted into the bloodstream where they become monocytes. Once monocytes exit the circulation and migrate into tissue, they differentiate into tissue specific resident macrophages, such as Kuppfer cells in the liver or alveolar macrophages in the lung [17]. These resident macrophages serve an immuno-surveillance function as a part of innate immunity, constantly eliminating foreign antigens from tissues and organs [18]. Alveolar macrophages, which are located at the air-tissue interface in the lung, are the initial cells that interact with inhaled microorganisms and particulate matter [19]. During normal respiration a human breathes in an estimated 7,000 liters of air per day [20]. Therefore, the alveolar macrophage's rapid recognition of invading pathogens via pattern recognition receptors (PRR) recognizing pathogen associated molecular patterns (PAMPS) and subsequent phagocytosis, is vital to maintaining healthy tissue [21]. Any impairment of these cells by PB1-F2 would greatly facilitate the establishment of a bacterial pulmonary infection. Also these resident macrophages communicate with the adaptive arm of the immune response via antigen presentation to CD4^+ ^T_*H *_cells. This antigen presentation results in clonal expansion of a given CD4^+ ^T_*H *_cell lineage and subsequently a range of downstream effector functions are induced, such as cytotoxic T lymphocyte (CTL) activation, antibody production, and inflammation [22]. By PB1-F2 specifically targeting macrophages for elimination, the induction of the acquired immune response is delayed and debilitated. This will allow for reduced clearance of Influenza A virus. A prolonged presence in the host will increase damage to host tissue and also produce more infectious virions that will enable increased transmission. More importantly, any delay or impairment in the immune response by PB1-F2 mediated destruction of professional antigen presenting cells could facilitate an opportunistic bacterial infection.

**Figure 1:**
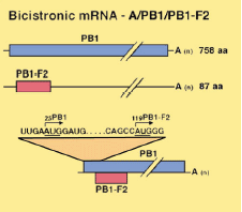
**The bisistronic PB1 gene segment of Influenza A Virus**. Here we see the two open reading frames of the PB1 gene segment of Influenza A Virus. The red segment corresponds to the alternate reading frame that encodes the PB1-F2 protein whose start site is 120 bp downstream of the PB1 polymerase gene. This figure was adapted form Lamb et al. 2001 [23].

The initial discovery of the PB1-F2 protein by Chen et al. occurred via a broad search for antigenic Influenza A viral peptides that are encoded by alternate reading frames. Chen et al. also examined if these novel polypeptides are presented by major histocompatibility complex I and in turn recognized by CD8^+ ^CTL on the surface of infected cells. After scanning the Influenza A genome for this novel antigenic, CTL-activating peptide, it was found to correspond to a protein encoded by an alternate reading frame found within the PB1 gene segment [2]. A simple schematic representation of the PB1-F2 can be seen in Fig. 1 adapted from Lamb et al. 2001. It is believed this alternate reading frame initiates at a start site (+) 120 base pairs upstream from the PB1 gene reading frame and is expressed possibly due to ribosomal scanning [23]. Ribosomal scanning is the process by which the 40S subunit of the ribosome, after loading directly downstream of the 5' cap by the translation machinery, "scans" for an AUG triplet to initiate translation; however if the ribosome recognizes an AUG in an alternate reading frame, a novel polypeptide could be produced [24]. When analyzing the PB1-F2 protein, it was found to be rather short lived in the replication cycle, with its maximum expression occurring approximately 5 hours post-infection [2]. The protein was found to localize to the inner and outer membranes of mitochondria via a basic amphipathic helix in its C-terminus [25]. When localized to the mitochondria, PB1-F2 can induce a dramatic degradation of mitochondrial morphology, which results in the reduction of the membrane potential and the induction of apoptosis [26]. PB1-F2 possibly functions by creating nonspecific pores in lipid bilayer membranes [27]. Interestingly this apoptotic induction occurs in monocytes much more readily than in epithelial cells, which was confirmed experimentally in tissue culture. This apoptotic induction can occur both when the protein is expressed intracellularly or simply present extracellularly to the macrophages [23]. It has also been shown that this PB1-F2 protein is not required for replication due to the viability in tissue culture of knockout viruses lacking the PB1-F2 reading frame [1]; interestingly the PB1-F2 knockout strain did show a marked decrease, approximately 50%, in their ability to induce monocyte apoptosis.

In sum, these *in vitro *findings strongly suggested that the presence of the PB1-F2 protein would have an increasing effect on viral pathogenicity *in vivo*. This hypothesis was confirmed by a recent study conducted by Zamarin et al., which examined PB1-F2's contribution to Influenza A virus pathogenesis in a mouse model. Indeed a virus expressing the PB1-F2 was found to have an increased virulence when compared to a knockout virus lacking the alternate reading frame. Also the presence of higher viral titers in the lungs of mice infected with a PB1-F2 expressing strain demonstrated a reduction in the animal's ability to clear the virus as well as mount an effective immune response [1]. Since PB1-F2 is found to have an apoptotic effect on professional antigen presenting cells, the PB1-F2 mediated killing of these cells could impede antigen presentation to the adaptive arm of the immune response, thus allowing for the increased pathogenicity of the virus.

Interestingly this alternate reading frame is found in the pandemic strains of 1968, 1957, and the infamous 1918 strain [1]. Indeed these viruses have a markedly pathogenic phenotype evident by the staggering death rates associated with each pandemic strain. All previous conclusions suggest that the increased pathogenicity of viruses encoding this protein is solely due to the virus's ability to reduce its own antigen presentation as well as reduce viral clearance. However, it is known that the major cause of death associated with an Influenza A virus infection is the establishment of a secondary bacterial infection [9]. Granted the cytotoxic, primary Influenza A infection is vastly damaging and PB1-F2 contributes to viral virulence by inducing apoptosis in macrophages. This targeted elimination of macrophages increases virulence by reducing antigen presentation and preventing crosstalk between the innate and adaptive arms of the immune system. However, the pathogenic enhancement of the PB1-F2 expressing viruses may also be due to an infected host's increased susceptibility to a secondary bacterial infection. This is an intriguing possibility and one that has been overlooked by previous studies.

## Conclusion

Currently it has been found after large-scale, exhaustive sequence analysis of avian Influenza A virus isolates that the PB1-F2 transcript is under the highest positive selective pressure for nonsynonymous substitutions [28]. This combined with the emerging threat of a possible human pandemic of Influenza A virus brings the enhancement of pathogenicity by PB1-F2 to the forefront. The World Health Organization's (WHO) policy now is to recommend the stockpiling of antibiotics to combat secondary bacterial infections associated with an outbreak of pandemic Influenza A virus [29]. If one could also develop an antiviral antagonist of the PB1-F2 protein (especially due to its ability to induce macrophage apoptosis when present extracellularly) the targeted destruction of professional antigen presenting cells could be inhibited. Therefore, the ability to clear virus and more importantly to fend off opportunistic bacterial infections would be maintained. The inhibition of PB1-F2 could prove to have a profound effect on human health because this could reduce the high rates of mortality associated with pandemic and epidemic Influenza A viruses carrying this alternate reading frame.
